# Genetic characteristics and molecular epidemiology of vancomycin-resistant *Enterococci* isolates from Caribbean countries

**DOI:** 10.1371/journal.pone.0185920

**Published:** 2017-10-11

**Authors:** Patrick Eberechi Akpaka, Shivnarine Kissoon, Padman Jayaratne, Clyde Wilson, George R. Golding, Alison M. Nicholson, Delores B. Lewis, Sandra M. Hermelijn, Alexis Wilson-Pearson, Ashley Smith

**Affiliations:** 1 The University of the West Indies, Paraclinical Sciences, St. Augustine, Trinidad & Tobago; 2 McMaster University, Department of Pathological Sciences, Hamilton, Canada; 3 King Edward VII Memorial Hospital, Department of Pathology and Microbiology, Hamilton, Bermuda; 4 Public Health Agency of Canada, National Microbiology Laboratory, Winnipeg, Canada; 5 The University of the West Indies, Department of Microbiology, Mona—Kingston, Jamaica; 6 The University of the West Indies, Faculty of Medical Sciences, Cave Hill, Barbados; 7 Academic Hospital Paramaribo, Medical Microbiology Laboratory, Paramaribo, Suriname; 8 Georgetown Public Hospital Corporation, Microbiology Laboratory, Georgetown, Guyana; 9 King Edward VII Memorial Hospital, Department of Pharmacy, Hamilton, Bermuda; Ross University School of Veterinary Medicine, SAINT KITTS AND NEVIS

## Abstract

Emergence of vancomycin-resistant Enterococci (VRE) that first appeared on the stage about three decades ago is now a major concern worldwide as it has globally reached every continent. Our aim was to simply undertake a multinational study to delineate the resistance and virulence genes of clinical isolates of VRE isolates from the Caribbean. We employed both conventional (standard microbiological methods including use of E-test strips, chromogenic agar) and molecular methods (polymerase chain reactions–PCR, pulsed-field gel electrophoresis–PFGE and multilocus sequence typing–MLST) to analyze and characterize 245 Enterococci species and 77 VRE isolates from twelve hospitals from eight countries in the Caribbean. The PCR confirmed and demonstrated the resistance and virulence genes (*van*A and *esp*) among all confirmed VRE isolates. The PFGE delineated clonally related isolates from patients from the same country and other countries in the region. The main sequence types of the VRE isolates from the region included STs 412, 750, 203, 736 and 18, all from the common ancestor for clonal complex 17 (CC17). Despite this common ancestor and association of outbreaks of this lineage clones, there has been no reports of outbreaks of infection by VRE in several hospitals in the Caribbean.

## Introduction

Vancomycin-resistant Enterococci (VRE) species notably *E*. *faecalis* and *E*. *faecium* are the two most common enterococci pathogens associated with numerous human infections including urinary tract infections, intra-abdominal, pelvic, soft tissue infections, bacteremia and endocarditis [1]. These species have been recognized as important human pathogens worldwide. In the United States they are the most common cause of nosocomial infections but second in ranking to Staphylococci species in healthcare facilities [2]. Spread of infections by these VRE species have now reached global proportions [3] since they were first reported in the United Kingdom and France in 1988[4, 5], North America in 1993 [6], and Asia with its first report in 1994 in Singapore [7].

In the Caribbean, there is paucity of data or information regarding types, prevalence or genetic characteristics of VRE infections or even resistance genes, clones or sequence types of vancomycin resistant isolates from the different countries. Some data is available on VRE infections and isolates from their North and South American neighbors (Mexico, Argentina, Brazil, Colombia, Ecuador, Paraguay, Peru and Venezuela) [8–11]. Few reports have recently been published to highlight VRE infections in some of the Caribbean nations [12, 13] but generally there is still a gross under representation of published data from these countries.

Thus, this multinational study was undertaken to investigate the antimicrobial resistance patterns, clonal relationships, virulent factors (*esp* and *hyl* genes) and population genetics of VRE isolates recovered from twelve hospitals in eight Caribbean nations. To the best of our knowledge, this is the first time such multinational report on VRE in the Caribbean is being undertaken.

## Materials and methods

### Participating countries and data

Twelve hospitals from eight countries (Barbados, Bermuda, Guyana, Jamaica, St. Lucia, St. Vincent, Suriname and Trinidad & Tobago), participated in the study. Clinical specimens were processed at hospitals from the different countries and bacteriology of vancomycin-resistant enterococci diagnostics were performed at each of the hospitals from these participating countries according to validated local protocols including either automated methods (Vitek or MicroScan system) or manual methods. Isolates from participating countries were from routine clinical specimens that included blood, peritoneal fluid, wound swabs (abscesses, surgical wounds, joint aspirates) and urine.

At the participating hospital or country, a standardized questionnaire was also used to collect basic demographic and clinical information on the patient including site of infection, age, ward, gender, risk factors (such as co-morbid condition, hospitalizations, previous antibiotic consumption or antibiotic treatment), colonization or infection and patient’s outcome. All participating countries followed the study protocols and did not send any duplicate isolates from the same patient or isolate deemed to be from colonization sites.

These confirmed and presumptive VRE isolates from the participating countries were then shipped by courier to the collection center: Microbiology/Pathology Unit, Department of Paraclinical Sciences, The University of the West Indies, Trinidad and Tobago for further analysis. The VRE isolates were received from participating countries in the Caribbean at different times during the period August 2009 to July 2014 at the collection center in a nutrient agar transport medium. Each time the isolate is received (once from each participating hospital) they are stored in minus 70^°^C until further analysis. At this laboratory Unit in Trinidad and Tobago, the purity of the isolates was determined using standard laboratory methods.

### VRE screening and confirmation

Screening for vancomycin resistance was performed using brain heart infusion agar (DIFCO), Bile esculin azide agar (BEAA, Oxford) containing 6mg/L of vancomycin. Two types of chromogenic agar plates [CHROMagar VRE (CHR) medium (CHROMagar, Paris, France) and ChromID VRE (C-ID) medium (bioMerieux, France) were used to phenotypically confirm the vancomycin resistance between the Enterococci species following the production of two different colony colors after incubation [14].

All tests were validated using quality control strains. Positive controls, *E*. *faecium* ATCC 700221 (mauve) and *E*. *faecalis* ATCC 51299 (green) and negative controls *Escherichia coli* ATCC 25922 (no growth) and *E*. *faecalis* ATCC 29212 (no growth).

### Antimicrobial susceptibility testing

The *in vitro* activity of ampicillin, chloramphenicol, ciprofloxacin, daptomycin, erythromycin, gentamicin, levofloxacin, linezolid, penicillin, quinupristin/dalfopristin, tetracycline, teicoplanin, tigecycline and vancomycin were evaluated against these non-repeat clinical isolates of vancomycin resistant *E*. *faecium* and *E*. *faecalis* by minimum inhibitory concentration (MIC) tests with Epsilometer (E-test) strips (AB Biodisk, Solna, Sweden). The E-test was performed on Mueller Hinton agar supplemented with 50 mg/l calcium (Difco, USA) and MIC values were interpreted according to the CLSI guidelines [15]. The MIC value for tigecycline was interpreted using the EUCAST guidelines [16].

### Determination of glycopeptide resistant genes

All isolates (n = 245) were shipped to McMaster University Canada, where they were all conventionally identified as VRE at the species level using multiplex polymerase chain reaction (PCR), as previously described by Jayaratne and Rutherford with modifications [17]. Briefly, prepared bacteria cells in normal saline mixed in lysis buffer were subjected to PCR amplification in 25 μL reaction mixtures containing deoxynucleoside triphosphate, two primers (*van*A: forward, 175-GGGAAAACGACAATTGC-191; reverse, 907-GTACAATGCGCCGTTA-891; *van*B: forward, 173-ATGGGAAGCCGATAGTC-189; reverse, 807-GATTTCGTTCCTCGACC-791), Taq polymerase, MgCl_2_, buffer and H_2_O. The samples were subjected to 30 PCR cycles, each consisting of 30 seconds of denaturation at 94^°^C, one minute of annealing at 54°C, and one minute of elongation at 72°C. PCR products were analyzed by electrophoresis on 1% agarose gels and were stained with ethidium bromide.

The *van*A and *van*B genes were also detected using the multiplex Loop-mediated isothermal amplification (mLAMP) as was previously described [18]. A *van*A strain (*E*. *faecium* ATCC 700221), a *van*B strain (*E*. *faecalis* ATCC 51299), and a vancomycin susceptible *E*. *faecalis* (ATCC 29212), 16S rDNA internal PCR amplification control, were run with each set of reactions as quality positive and negative controls.

### Detection of *esp* and *hyl* genes by PCR

The presence of *esp* and *hyl* genes were tested for in all the Enterococci isolates (n = 245) by methods previously described by Vankerckhoven et al. [19].

### Pulsed-field gel electrophoresis

Based on standard tests (Chromogenic agar and E-test analysis); and molecular analysis (PCR and mLAMP) only 77 isolates were completely confirmed as VRE and were subjected to pulsed-field gel electrophoresis (PFGE) by the methods described by Murray et al. and Turbelidze et al. with some modifications [20, 21]. Results of the DNA banding patterns were interpreted by visual inspection, according to the criteria specified by Tenover et al. [22].

### Repetitive-sequence-based-PCR (Rep-PCR)

Repetitive-sequence-based-PCR (Rep-PCR) method was also used to further characterize the isolates as described by Healy et al. [23]. This is a rapid typing procedure that amplifies the regions between the noncoding repetitive sequences in bacterial genomes [24].

DNA was extracted using a one μL loop of plated culture or one mL of broth culture and the Ultraclean Microbial DNA Isolation Kit (Mo Bio Laboratories, Solana Beach, Calif.) following the manufacturer’s instructions. The extracted DNA was amplified using the DiversiLab Enterococcus fingerprinting (Spectral Genomics, Inc., Houston, TX) according to the manufacturer’s instructions. Genomic DNA, the Rep-PCR primer (Enterococcus) AmpliTaq, and PCR buffer (Applied Biosystems) were all mixed together and subjected to thermal cycling. Amplicons were separated by 1.5% agarose gel electrophoresis (gels, 25 by 25 cm2) containing ethidium bromide (3 μg/mL in gel and in 1x tris-acetate-EDTA running buffer) for six hours at 120V in a recirculating electrophoresis unit. DNA ladder (Promega, USA) was used as DNA size markers. Gel images were captured on the Gel Doc imaging system using Quality One Software version 4.4.1 (Bio-Rad Laboratories, Hercules, CA, USA).

### Multilocus sequence typing (MLST)

Multilocus sequence typing (MLST) was performed on representatives of the VRE isolates (n = 30) selected based on their pulsed-field gel electrophoresis profiles, countries and body sites. The sequencing typing for the *E*. *faecium* isolates was carried out according to procedures previously reported in literature by Homan et al. [25].

For the sequence typing of vancomycin-resistant *E*. *faecalis*, these were performed according to primers and procedures established by Ruiz-Garbajosa et al. [26]. PCR amplicons were sequenced using a BigDye Terminator v1.1 cycle sequencing kit (Life Technology, CA, USA) on an ABI 3500xl genetic Analyzer.

MLST sequences were then queried into the MLST databases, that is (http://efaecium.mlst.net/) and (http://efaecalis.mlst.net/) to determine their sequence types (STs). Their genetic relatedness was explored using goeburst [27].

### Statistical analysis

Data was analyzed using a statistical package for social science (SPSS) version 20. Statistical evaluations were carried out at 95% CI and p-value <0.05 was considered as significant.

### Ethical approval

This study was approved by the Campus Ethics Committee of the University of the West Indies, St. Augustine, Trinidad and Tobago. Permission to collect the isolates at the various hospitals were also obtained where necessary. Consents from patients were not obtained as their records and information were anonymized and de-identified prior to analysis.

## Results

A total of 155 Enterococcus species isolates (mainly *E*. *faecium*) were received from seven Caribbean countries. Ten isolates were excluded in the initial analysis due to protocol violations (contamination, source and basic demographic data were not included in the protocol). There were 100 isolates from Trinidad and Tobago—giving a total of 245 isolates that were analyzed. Some other species from Trinidad and Tobago isolates such as *E*. *casseliflavus*, *E*. *gallinarum*, *E*. *hirae* and *E*. *durans* were excluded from the analysis. Breakdown of isolates from all countries included in the final analysis were as follows: Trinidad and Tobago 40.8% (n = 100), Suriname 12.6% (n = 31), Barbados 9.8% (n = 24), Guyana 8.6% (n = 21), St. Vincent 7.7% (n = 19), Bermuda 7.3% (n = 18), St. Lucia 6.9% (n = 17) and Jamaica 6.1% (n = 15).

Of all these 245 Enterococci species from the participating countries in this study, vancomycin-resistant enterococci (VRE) accounted for 31.4% (77/245); and these VRE isolates (n = 77) were included in the final molecular analysis. The breakdown of their clinical sites and countries of origin are depicted in Table 1 below. Majority were *E*. *faecium* species 90.9% and the rest were *E*. *faecalis* 9.1% (obtained only from hospitals in Trinidad & Tobago). More than half of these VRE isolates were from females 58% (42/77); and whilst the majority 52% (40/77, p = 0.05) of the isolates were recovered from urine samples, the least 5.2% (4/77) were from blood and peritoneal fluid respectively. All the 77 VRE isolates (100%) were noted to have been recovered from hospitalized patients; and thus represented healthcare associated isolates and infections. The patients whose clinical specimens yielded these isolates fulfilled the criteria of developing their infections 72 hours or more after being admitted into hospital. The distribution of the hospital facilities where these patients were treated in the countries are depicted on Table 1 and reveals that there were almost equal distributions of these isolates in several disciplines (surgery, medicine or intensive care units) of the hospital facilities.

**Table 1:** Distribution of the clinical specimens and hospital facilities of VRE isolates from Caribbean countries.

Country	N	Species		Clinical source				Hospital facility			
		Efm	Efc	B	WS	U	GIT	SUR	MED	ICU	BU
Jamaica	8	8	0	0	3	5	0	1	0	7	0
St. Vincent	1	1	0	0	1	0	0	0	0	1	0
Suriname	1	1	0	0	0	1	0	0	0	1	0
Guyana	5	5	0	2	1	1	1	2	0	3	0
Barbados	4	4	0	0	1	3	0	1	1	2	0
St. Lucia	1	1	0	0	0	1	0	1	0	0	0
Bermuda	12	12	0	1	4	6	1	3	4	5	0
Trinidad & Tobago	45	38	7	1	19	23	2	14	24	6	1
Total	77	70	7	4	29	40	4	22	25	25	1

N–number of isolates included in analysis; Efm–*E*. *faecium*; Efc–*E*. *faecalis*; B–Blood; WS–wound swab; U–urine; GIT–gastrointestinal tract; SUR–Surgical ward; MED–Medical ward; ICU–Intensive Care Unit; BU–Burns unit

Most of the VRE isolates were obtained from patients in the age group 50–59 and least from 0–9 years age group (Result not shown). The median age of 50 (range 9–91) was observed among the patients with VRE infections. Analyzed risk factors in patients of these isolates revealed that those who are diabetics 39% (30/77, p = 0.04), hypertensive 31% (24/77, p = 0.03), cardiovascular diseases 29% (22/77, p = 0.04) and respiratory diseases 5.1% (4/77, p = 0.06) were encountered in that order. There were also some patients who had combination of diseases but these were not significant when analyzed.

The susceptibility profiles of the VRE isolates are depicted on Table 2. All Enterococci isolates confirmed as VRE had a MIC value for vancomycin ≥ 32μg/mL. These VRE isolates were also noted to be 100% susceptible to daptomycin (MIC≤4ug/ml), linezolid (MIC≤2ug/ml), quinupristin/dalfopristin (MIC≤1ug/ml), teicoplanin (MIC≤6μg/mL), tigecycline (MIC≤4ug/ml), and 94% to gentamicin and 100% resistant to ciprofloxacin, erythromycin, levofloxacin. The susceptibility profiles of the seven vancomycin resistant *E*. *faecalis* from Trinidad and Tobago showed that they were all resistant to quinolones, macrolides, but completely sensitive to daptomycin, linezolid, quinupristin/dalfopristin, teicoplanin and tigecycline.

**Table 2:** Antibiotic susceptibility profile of clinical vancomycin resistant Enterococci (VRE) isolates from Caribbean countries, 2009–2014 (%).

Antimicrobial agent	*E*. *faecium*		*E*. *faecalis*	
	Sensitive	Resistant	Sensitive	Resistant
Ampicillin	5 (7.1)	65 (92.8)	0	7 (100)
Chloramphenicol	15 (21.4	55 (78.6)	2 (28.6)	5 (71.4)
Ciprofloxacin	0	70 (100)	0	7 (100)
Daptomycin	70 (100)	0	7 (100)	0
Erythromycin	0	70 (100)	0	7 (100)
Gentamicin	65 (92.8)	5 (7.2)	4 (57.1)	3 (42.9)
Levofloxacin	5 (7.1)	65 (92.9)	0	7 (100)
Linezolid	70 (100)	0	7 (100)	0
Penicillin	0	70 (100)	0	7 (100)
Quinuprustin/Dalfopristin	70 (100)	0	7 (100)	0
Streptomycin	45 (64.3)	25 (35.7)	5 (71.4)	2 (28.6)
Tetracycline	25 (35.7)	45 (64.3)	0	7 (100)
Teicoplanin	70 (100)	0	7 (100)	0
Tigecycline	70 (100)	0	7 (100)	0
Vancomycin	0	70 (100)	0	7 (100)

All the *E*. *faecium* isolates possessed the *van*A genes, while all the *E*. *faecalis* (only from Trinidad and Tobago) possessed the *van*B genes. Overall, the *esp* genes were detected in all (100%) VRE isolates. None of the isolates had *hyl* genes. The most prevalent *esp* profile was A6-C5 (18/70, 25.7%) followed by A5-C6 (6/70, 8.8%), A6-C3 (6/70, 8.8%), A4-C5 (18/70, 25.7%), A5-C7 (10/70, 14.3%), A3-C6 (12/70, 17.1%). The analysis of molecular typing demonstrated six PFGE patterns (Table 3) among the 70 vancomycin resistant *E*. *faecium* and seven *E*. *faecalis* isolates. The predominant clones were one and four (PFGE-1 and PFGE-4) and each clone occurred in 25.7% (18/70) isolates respectively. Clone one was present in four countries: Jamaica (n = 5), St. Vincent (n = 1), Guyana (n = 1) and Trinidad & Tobago (n = 11). Clone four was present in Barbados (n = 1), St. Lucia (n = 1), Guyana (n = 2), Bermuda (n = 4) and Trinidad & Tobago (n = 10). Clones two and three (PFGE-2 and PFGE-3) were represented by six isolates each, PFGE-2: Guyana (n = 2), Barbados (n = 2), Bermuda (n = 2) and PFGE-3: Trinidad & Tobago (n = 2), Jamaica (n = 2) and Barbados (n = 2). Clones five and six (PFGE-5 and PFGE-6) had 14.3% (10/70) and 17.1% (12/70) isolates respectively. All the seven vancomycin-resistant *E*. *faecalis* had an identical PFGE pattern indicating they belong to the same clone; and they were all recovered from urine samples of patients in Trinidad and Tobago hospitals.

**Table 3:** Distribution of the virulent genes, PFGE patterns and sequence types of VRE isolates from Caribbean countries.

Country	N	Species		Virulent genes				PFGE profile						Sequence Types				
		Efm	Efc	*esp*	*hyl*	*van*A	*van*B	1	2	3	4	5	6	18	203	412	736	750
Jamaica	8	8	0	8	0	+	0	5	0	2	0	1	0				+	
St. Vincent	1	1	0	1	0	+	0	1	0	0	0	0	0			+		
Suriname	1	1	0	1	0	+	0	0	0	0	0	0	1			+		
Guyana	5	5	0	5	0	+	0	1	2	0	2	0	0			+		
Barbados	4	4	0	4	0	+	0	0	2	2	0	0	0				+	
St. Lucia	1	1	0	1	0	+	0	0	0	0	1	0	0				+	
Bermuda	12	12	0	12	0	+	0	0	2	0	4	0	4	+			+	+
Trinidad & Tobago	45	38	7	45	0	+	0	11	2	2	11	7	7		+	+	+	
Total	77	70	7	77	0	77	0	18	8	6	18	8	8					

N–number of isolates included in analysis; Efm–*E*. *faecium*; Efc–*E*. *faecalis*; *esp*–Enterococcal surface protein; *hyl*–hyaluronidase; *van*A–Vancomycin resistant A gene; *van*B—Vancomycin resistant B gene

MLST was performed for selected VRE isolates (n = 30); twenty-seven *E*. *faecium* and three *E*. *faecalis* to determine their sequence types. The isolates were selected based on their PFGE profiles. As depicted on Table 3, the results revealed that the *E*. *faecium* isolates belonged to 5 main sequence types (STs): ST412 (44.4%, 12/27), ST736 (40.7%, 11/27), ST203 (3.7%, 1/27), ST18 (7.4%, 2/27) and ST750 (3.7%, 1/27), while the *E*. *faecalis* isolates belonged to the ST6. The ST203 and ST750 are single locus variants (SLV) of ST412 while ST736 is a triple locus variant (TLV) of ST412 and ST18 is a quadruple locus variant (QLV) of ST412. The ST412 was found in Guyana, Trinidad & Tobago, Suriname and St. Vincent. ST736 was found in Jamaica, Barbados, Bermuda, St. Lucia and Trinidad & Tobago., ST203 was found in Trinidad & Tobago while ST750 and ST18 were found in Bermuda. The MLST data suggested that all *E*. *faecium* isolates in this study belong to one clonal complex 17 (CC17). Strains that had the allele of the purK gene have been reported to be related to the epidemic vancomycin-resistant *E*. *faecium* strains [28, 29].

## Discussion

The genetic features and molecular epidemiology of VRE isolates associated with infections from several hospitals and countries in the Caribbean is being reported for the first time. This analysis revealed that the prevalent glycopeptide resistance in VRE clinical isolates from all the countries were mediated by the *van*A genes. Although our analysis tested for only two genes—*van*A and *van*B of the nine vancomycin resistant cluster genes (*van*A to *van*N), interestingly, only *van*A was identified in all the vancomycin resistant *E*. *faecium* isolates. This is not surprising, since several *E*. *faecium* isolates studied in the region (North and South American countries) have all reported predominance of *van*A [6, 8–11, 30]. Besides, the van*A* cluster is the most commonly acquired mediator of vancomycin resistance in enterococci species [31] and there have been reports of its ubiquitous nature, usually associated with mobile genetic elements (MGE) such as transposons and highly associated with several environmental sources [32].

In our analysis of VRE isolates from the Caribbean region, there was a high rate of ampicillin and quinolones (ciprofloxacin and levofloxacin) resistance respectively. Several other studies have reported similar resistance to several agents including ampicillin, aminoglycosides and quinolones [30, 31]. In Trinidad and Tobago, the high level of quinolones resistance among its VRE isolates could be attributed to injudicious and over use of this class of antibiotics as they are sold over the counter without restrictions or prescriptions in the country. The reason for a high rate of VRE resistance to these drugs is not known in the other countries. Also, unlike reports elsewhere [30, 32], our analysis reveals complete susceptibility to daptomycin, linezolid, tigecycline, quinupristin/dalfopristin and teicoplanin among all VRE (both *E*. *faecium* and *E*. *faecalis*) isolates from the region. This implies that whereas there are limited treatment options in several places for VRE infections; a challenge to many clinicians, there are many available treatment choices or options in the region.

Similar to reports of vancomycin-resistant *E*. *faecium* in some Latin American countries [8–11], our Caribbean isolates expressed full *esp* virulence genes described as a marker of distinct genetic lineage of vancomycin-resistant *E*. *faecium* spreading in hospitals. As reported by Billsstrom *et al*., a significant correlation was found between the presence of *esp* in vancomycin-resistant *E*. *faecium* and resistance to ampicillin and ciprofloxacin [33]. We could therefore hypothesize that since all our analyzed isolates were from hospital associated infections and resistance to ampicillin and quinolones, these could have been promoted by their possessing the *esp* genes.

None of the VRE isolates from the Caribbean countries possessed the *hyl* gene that has been reported to belong to the glycoside hydrolases, which is conserved among enterococci involved with nosocomial infections such as bacteremia [34]. Isolates producing the *hyl* gene have been widely detected in vancomycin-resistant *E*. *faecium* isolates from Brazil and the USA, where they are reported in cases associated with VRE outbreaks as well as in multidrug resistant infections [35, 36]. There has never been serious reports of VRE outbreaks in any of the hospitals in the region (except in Jamaica that experienced an outbreak in the ICU at one of its hospitals over ten years ago); besides, none of the VRE isolates we analyzed were multidrug resistant.

The PFGE and MLST tools were very useful in analyzing some of the specific characteristics of the VRE isolates from the Caribbean countries. The PFGE profiles indicated that there were some clonal relationships between some vancomycin resistant *E*. *faecium* isolates from different patients in the same hospitals, as well as some isolates between the different countries. This could very well suggest horizontal transmission through hospitalized patients or even healthcare workers or other people. The MLST analysis assisted so much in determining ancestry of the isolates by revealing that the prevalent vancomycin-resistant sequence types of the *E*. *faecium* isolates were STs (412, 750, 203, 736 and 18). All these STs have been linked to the clonal complex 17 which has a worldwide distribution and is common within North and South American regions as has previously been reported by several other workers [3, 8, 9, 11, 34, 35].

Limitations of this ambitious and humble task will not be overlooked. The small number of the VRE isolates in this analysis could be very insufficient to generalize conclusions about the prevalence or genetic characteristics of VRE isolates in the Caribbean countries. Nevertheless, this data constitutes an important contribution to the plethora of published information on VRE molecular genetics, especially considering the fact that most reports originate from several Latin American countries and several developed countries but data from the Caribbean countries are underrepresented. Baseline data from the Caribbean has been put forward and there is still much to be done to increase our knowledge of VRE evolution in this part of the world.

## Conclusions

Vancomycin-resistant *E*. *faecium* isolates from the Caribbean countries are not multidrug resistant and vancomycin resistance appears to be mediated mainly by the *van*A gene. The infections associated with the isolates in the region appears to be hospital associated. Although none of the isolates harbors *hyl* but mainly the *esp* gene involved in outbreaks, there has never been any report of outbreaks of VRE infections in the region. Predominant sequence types STs in the region include STs 412, 750, 203, 736 and 18. All these STs are from the clonal complex 17 (CC17) ancestor. This is similar to clonal complexes also circulating in the Americas.
